# Health Policy Development During COVID-19 in Saudi Arabia: Mixed Methods Analysis

**DOI:** 10.3389/fpubh.2021.801273

**Published:** 2022-03-14

**Authors:** Wadi B. Alonazi, Eman A. Altuwaijri

**Affiliations:** ^1^Health Administration Department, College of Business Administration, King Saud University, Riyadh, Saudi Arabia; ^2^Department of Administrative and Human Sciences, College of Applied Studies and Community Service, King Saud University, Riyadh, Saudi Arabia

**Keywords:** health management, COVID-19, document analysis, public health, authority

## Abstract

Healthcare systems are increasingly required to utilize effective approaches, apply evidence-based practice, and consequently sustain successful strategic management. Document analysis provides insights into the effective management tools applied by agencies to respond to crises. This article provides a practical exploration of how the Saudi health authority applied effective measures to eventually reduce the administrative and clinical consequences while managing the COVID-19 pandemic. The conceptual descriptive framework was based on health policy triangle of Walt and Gilson. Official reports and supporting documents issued by the Saudi government toward COVID-19 were operationally analyzed. Moreover, five healthcare professional experts were invited in a semistructured interview to assess the strategic steps that have been utilized to minimize the health risk by conducting a healthcare risk analysis. Various documents showed that two major entities were responsible for managing regulations and medications of COVID-19 in addition to six other entities that were partially involved. Although each entity was approved to work independently, their efforts were cohesively associated with each other. Most documents were well-applied on personal, social, organizational, and national strata. However, it is unclear how lessons identified became affirmative, while the collaboration remains vague, especially under the emergence of a new entity such as the Public Health Authority. Healthcare professional experts also positively supported the effectiveness of such policies to confront COVID-19 through the following three domains: health guidelines, utilizing simulation (telehealth/telecommunication) services, and ensuring continuity of services.

## Introduction

Late 2019, an infectious disease is known as corona virus disease (COVID-19) has speared widely all over the world causing devastating impact not only on global health but also on the world economy that reached its lowest levels. The first cases of pneumonia of unknown cause were detected in Wuhan, China. This has impacted China and countries of the world causing a global outbreak. Each country, as a result, initiated some effective measures to reduce the consequences of such a pandemic locally, or with the support of the World Health Organization (WHO) (1).

The kingdom of Saudi Arabia (KSA) has encountered various challenges during this pandemic but was able to set some measures to reduce the impact to the minimum. Protective measures include building COVID-19 healthcare centers, providing free treatment and healthcare to all, specifying fever clinics in all cities, public and private hospitals, and were designed only to receive patients with COVID-19 symptoms (2, 3). Yet, around 60,000 cases were reported in the early 6 months of the crisis, and the nation lost almost 8,000 lives by June 2021. Saudi Arabia, among many other international healthcare systems, has responded immediately to the pandemic by introducing various health and safety measures and precaution protocols, however, some of those systems often struggled to reduce the mortality and morbidity of COVID-19 among their population (4). Generally speaking, multiple factors are associated with the success in improving healthcare outcomes of healthcare policies, content, context, and processes (5). Moreover, KSA was among the early countries to mitigate the impact of COVID-19 even before the first case was reported. This was achieved by implementing early precautionary measures learned from past outbreaks, such as, the Middle East respiratory syndrome (MERS) and the severe acute respiratory syndrome (SARS) which are both classed as corona viruses. From early January 2020, such measures included the formation of a national committee to prepare for the possible introduction and spread of the virus and to follow up with global updates of the outbreak. Proactive decisions were announced by the committee that consisted of collaborated ministries like the ministry of health, education, interior, and more. Decisions included banning all flights from China, suspending entry to tourists and to those who were to visit the holy cities of Makkah and Madinah, and shifting education of all levels to online and virtual teaching (6). Hence, it would be academically and professionally valuable to assess the management of COVID-19 in the Saudi healthcare system during this pandemic.

### Defining COVID-19 Confirmed Cases

While there are three types of COVID-19 case definitions, namely, suspected cases, probable cases, and confirmed cases, ultimately, there are two major steps toward confirming cases and this may include:

1. Individuals who have symptoms and meet specific clinical criteria, like healthcare professionals, suspected individuals, and new-comers from overseas.
2. Results of testing of positive COVID-19 symptoms, through suspected or referral cases.

#### Healthcare Policy Analysis Model

Within the wide variety of models and theories, selecting the appropriate approach to analyze healthcare policies needs to clearly explain the domains that contribute insightfully to reducing the consequences of the phenomena. This model, for example, should be based on the research objectives and the availability of data within a particular context (7). However, analyzing the Saudi healthcare policy requires a comprehensive exploration of various factors and elements (8). The most widely, reliable, and valid framework to apply, in our case, is perhaps Walt and Gilson's healthcare policy triangle (9). This framework is particularly helpful in explaining the macro level of the healthcare policy and any other related politics (10). Simply, this triangular framework consists of: content, context, and process investigates through direct influencers to shape policy-making.

Walt and Gilson's triangular framework for healthcare policy is considered a useful tool to analyze healthcare policy systems due to their robustness and consistency (11). Contextual factors such as political, economic, and sociocultural influence the healthcare policy management of COVID-19, this is explained through a well-constructed control policy process of implementing, monitoring, and evaluation. Indeed, this model allows for a more objective analysis of previous healthcare policies, and it indicates a clear framework of how policy-making can make integration through actors, process, context, and content. Actors are defined as key players in formulating the influence on healthcare policy (12).

During COVID-19, those protocols were the most influential factors that significantly contributed to formulate the current medical guidelines. The process is a series of actions taken to document the experience of specific actions in a healthcare system from a research prototype into action in that system. Experimental results from the healthcare field environments are daily obtainable. While context is normally associated with facilities or barriers that may move or hinder healthcare outcomes, they may indicate failure or success on some occasions. Content is associated with variance, but tackling sources with heterogeneous grounded.

To simplify, the model includes the content, the context, and the process of policy reform conducted by the actors. Despite the interaction of each element with one another, the model will be clear through visualizing the risks enforced by many parties.

#### Risk-Adjusted Model

Maintaining services during normal activities is a critical step to the health risk adjustment, and this may include continuous monitoring to the delivery of safe and effective healthcare services (13). It is a reflection of how the system can survive to provide the basic and advanced medical services based on healthcare leadership responses to the crisis through effective measures. Statistics and health economics are tools widely used to measure adjusted risks. For example, daily reports of patients through monitoring structure, processes, and outcome activities are common to reduce health risks. This is normally associated with economic variables like cost-benefit analysis. Healthcare systems in KSA are diverse, however, the Ministry of Health (MOH) is considered the main healthcare provider that overlooks, regulates, and manages all the different healthcare systems in the country.

The COVID-19 pandemic has a major impact on healthcare systems across the globe and Saudi Arabia is not an exception. Saudi Arabia was one of the leading countries that initiated effective measures to reduce such risks. Those micro and macro levels were based on a series of applications and decisive measures of prevention, control, and specific treatments that were not fully documented in the literature (3, 14). The need to address changes in policies, personnel, and administrative preventive measures are key issues in public health and health risk adjustment. Indeed, rules and regulations were quickly stated and updated through a new design of the healthcare system delivery, representing interrelated groups (15).

Indeed, healthcare policies legislated by politicians, economists, and healthcare administrators interfere in collaboration to reduce the devastating impact of COVID-19 on the healthcare systems. The aim of this study is to analyze healthcare policies, on both the macro and micro levels in relevance to COVID-19 management in KSA using Walt and Gilson's policy triangular model (16).

## Methods

### Design

Qualitative and quantitative analysis design was used primarily to explore the initiatives proposed by the Saudi healthcare agencies to reduce the risk of COVID-19. The main research question is how the healthcare policy was influenced by certain factors while confronting the COVID-19 crisis. More than 106 official initiatives were analyzed. Incorporating policies, procedures, protocols, and guidelines, gray areas were combined with their associated main reference if there was any.

Expert healthcare professions represented in the sample were selected due to their healthcare performance that was linked to the pandemic outbreak. To explore themes, different techniques were used, for instance, word analysis and assimilating of texts and contexts.

### Procedure

In this analysis, this study explores the Saudi Arabian healthcare system in managing COVID-19 on macro and micro levels, supported by identified examples of relevant policy documents and interviews of the COVID-19 expert healthcare professional. Selected documents provided information about prevention, crisis control, and documents that were assessing the political, economical, social, and historical context of COVID-19 management in KSA. This study is a critical review based on various primary and secondary data collected from expertise, government references, reports, and articles.

The validity of the data was based on data credibility, transferability, dependability, and confirmability. Applying triangulation, through coding, analysis, and interpretation of official documents, was appropriately sufficient. Moreover, applying contextualization within multimeaningful channels assured inclusive data. Finally, data should be perpetually relevant to COVID-19 management policy with a significant influence on the population (17).

### Data Extraction and Synthesis

The primary aim of the document analysis was to categorize the contents, processes, and context of potential themes. Data search began with an examination of the clear-cut literature (government documents, official websites, and mass media websites). The published policy documents were indexed and listed based on a thematic paradigm. Each document was attributed to its main source, i.e., who issued it? Indeed, a neutral stance to obtain the data was applied to prevent bias. Initially, data were coded under multiple levels based on the level of influence allowing them to be recorded based on Walt and Gilson's policy triangular model.

Again, data were reclassified according to the study framework where the actors played three essential roles: content; context, and process. To do this, data were extracted in a template sheet based on the study framework. The study was limited to national healthcare initiatives by government policies and plans to minimize the risks of COVID-19.

### Data Collection

A semistructured interview was conducted independently and remotely via a teleconferencing software (Zoom) with five expert healthcare professionals to assess the following:

1. Was the Saudi healthcare system successful in managing the COVID-19 crisis? How and why?
2. Which entity/public organization was available 24/7 to reduce available risks? How?
3. To what extent do you believe that such a strategy has reduced available risks? Explain.
4. What are the major areas that the Saudi healthcare system targeted to reduce available risks? Give examples.
5. Which entity can continue more to support rules and regulations to manage the pandemic? How?

In addition, ten close-ended questions were asked to all five participants by an independent researcher from the Saudi Society of Health Administration. Participants had no previous contact with the interviewer. The interviewer followed specific interview guidelines that focused on the experiences and management of the participants during the COVID-19 pandemic. Direct and indirect inquiries were used to encourage participants to focus on certain aspects that needed to be explored in more analysis.

Interviews took place remotely during January and March of 2021 in Riyadh Region via teleconferencing software. All interviews were run in standard English, as participants were Western-country graduates with high proficiency in English. Each interview was around 30–45 min long. Experts were selected (purposeful sampling) based on their direct involvement in managing the crisis, first of all. This involved identifying and selecting subjects who are especially knowledgeable about COVID-19 and implementing its strategies in KSA.

### Data Analysis

In this stage, both themes and patterns were identified upon completing the research protocol transcription. Researchers then coded the data by ordering the basic theme code, then sketching the pattern coding.

Indeed, the sequential phases offered strata of triangulation based on the applied research model. Each step ensured alignment with the model, data accuracy, and replicability. The purpose of this step was to construct analysis rather than syntax analysis. All steps were based on deductive coding.

Utilizing quantitative data to measure various parameters was a helpful tool to explore more the initiatives proposed by gathered official documents. Thus, data gathered from national or international resources could be established across a population to infer a conclusion.

## Results

In an attempt to increase the number of documents and to ensure reliability, the researchers expanded the scope of the search for in-depth information from other official resources, as selected organizations are official national entities where daily surveillance of the pandemic is reported continuously. Essential guidelines and system implementations were found among eight institutions that confront COVID-19. Official resources included reports, guidelines, roles, and regulations, driven from the Ministry of Health (MOH), Ministry of Interior (MOI), Ministry of Education (MOE), Ministry of Human Resources (MOHR), Ministry of Finance (MOF), Ministry of Hajj and Umrah, Ministry of Information and Technology (MOIT), and finally the Ministry of Media (MOM).

Major and supportive entities involved in the formulation of the healthcare policy and engagement were also examined. Table 1 includes a summary of the volume of examined documents.

**Table 1:** List of public policy elements identified based on their influence.

**Level of policy engagement**	**Major entity**		**Supportive entity**					
	**MOH**	**MOI**	**MOE**	**MOHR**	**MOF**	**MOHU**	**MOI and T**	**MOM**
National	6^***^	6^***^	2^*^	5^**^	3^*^	4^*^	3^*^	3^*^
Organizational	6^***^	5^**^	3^*^	3^*^	2^*^	4^*^	2^*^	4^*^
Social	5^**^	5^**^	4^*^	2^*^	1^*^	2^*^	1^*^	4^*^
Personal	4^*^	6^***^	5^**^	1^*^	1^*^	1^*^	1^*^	2^*^

*** *High influence*;

** *Intermediate influence*;

* *Low influence (the number indicates the volume of distribution documents as a parameter)*.

As government reports were identified on two levels, only abstract judgments were reported. If no direct impact was observed; then the level of influence is secondary. After that, applying the deductive approach, the framework synthesis, then identifying and summarizing imperative data drawn from the document. First, each document was analyzed to express four levels: national, organizational, social, and personal. Such aim can thoroughly assess to what extent the elements of framework of the Walt and Gilson were reflected. Second, in a deductive approach, new themes were developed and included as they emerged from the data.

Simply, actors refer to key players who significantly contribute to developing, applying, managing, and accessing COVID-19 healthcare policies. This may include a wide range of organizations involved in significant activities that are likely to directly influence COVID-19 management.

On the micro level, the Saudi MOH is responsible for developing policies in which implementation programs support the system that is in alignment with regulations. As the MOH provides clinical services, the need to strictly support the administrative side is clear when there is a need to apply mandatory actions like lockdown, social distancing, and mask wearing.

Table 2 shows the sociodemographic characteristics of the expert healthcare professionals who participated in the study.

**Table 2:** Basic demographic characteristics of the experts (*n* = 5).

**Characteristics**	***n***	***%***
**Gender**		
Male	3	60
Female	2	40
**Education**		
Ph.D., or equivalent	2	40
Masters' degree	2	40
Bachelor degree	1	20
**Position**		
CEO	3	60
MD	1	20
Other	1	20
Experience	Min = 10	Max = 25

*CEO, chief executive office; MD, medical doctor*.

Table 3 describes basic inquiries of the process combat of the Saudi healthcare system toward COVID19 management from the point of view of participants.

**Table 3:** Close-ended interview questions result of selected expert healthcare professionals (*n* = 5).

**Question**	**Yes**		**Not sure**		**No**	
	***n***	**%**	***n***	**%**	***n***	**%**
Was the system effective?	5	100	0	0	0	0
Were all public institutions independent?	5	100	0	0	0	0
Was the system well integrated?	3	60	2	40	0	0
Was the risk reduced?	5	100	0	0	0	0
Was health leadership competent?	4	80	1	20	0	0
Were resources available?	3	60	1	20	1	20
Were national communications effective during the crisis?	3	60	1	20	1	20
Was there a national contingency plan?	4	80	1	20	0	0
Was there an organizational contingency plan?	2	40	1	20	2	40
Do you have an established risk modeling culture?	1	20	1	20	3	60

Experts who were interviewed as participants, also positively supported the effectiveness of introduced policies to confront COVID-19 through three domains: health guidelines, utilizing simulation (telehealth/telecommunication) services, and ensuring continuity of services. The following will discuss each theme individually.

### Health Guidelines

Health guidelines refer to both documentation and implementation of procedures to reduce risks among the population. Health guidelines emerged as a prominent theme from the semistructured interview data. The analysis indicated the perceptions of the expert healthcare professionals and understanding of the well-supported medical and non-medical guidelines. The essential step toward guidelines was patient access to healthcare services regardless of gender, ethnicity, and nationality. Immigrants and illegal residents were also allowed to be treated in public hospitals without any financial liabilities. Accessibility of records and information to track vulnerable cases were available to healthcare policymakers.

“I wouldn't be able to appreciate the ability of the public services, especially Public Health Authority, to confront COVID-19 till I received health guidelines to what to do, when, how, and why. Nothing better than guidelines that could confront the crisis.” (Expert healthcare professional 2)

Seniors, people with chronic disease, and healthcare staff had direct and quick access to healthcare services (18). All health professionals were strictly warned to apply all COVID-19 management protocols. Any violations will be assessed and may lead to penalties and law breaching.

“What the public policy added to healthcare system was medical guidelines including patient safety and high quality of care. Imagine we control COVID-19, but in unsafe environment? All our health plans will be invalid.” (Expert healthcare professional 5)

Thus, a subset of participants across all disciplines appreciated the advanced guidelines with its integrated system to reduce risks (19).

### Utilizing Simulation Services (Telehealth/Telecommunication)

The ability to solve real-world problems in a safe, efficient, and effective way is an essential element to utilize services. For instance, during the partial lockdown, many patients were unable to move freely to be examined by a physician. Virtual clinics were essential parts not only within remote areas but also in cosmopolitan cities.

“We are now launching a virtual hospital to be part of the services provided to the population. MOH will not stop improving the performance of healthcare services. All hospital facilities, including lab tests and x-rays will be monitored and operated in very innovate ways.” (Expert healthcare professional 1)

“Can you imagine that we are now using more than (20) health applications to reduce time and efforts for health providers and patients? The technology used is not reliable only but secure and other phases will be utilized to launch a virtual healthcare system.” (Expert healthcare professional 2)

“Online learning and education increased retention of information, changed dramatically, during COVID-19, with the distinctive rise of e-learning, whereby knowledge-transfer has become the value of education.” (Expert healthcare professional 5)

With this strategic shift, the Saudi healthcare system adopted simulations (telecommunication methods) in education, particularly in health schools, and continue to do so post-pandemic. Moreover, new paradigms evolved to impact not only providers of the healthcare services but also end-users of the system (21).

### Ensuring Continuity of Services

This sets out how healthcare organizations prepare for a pandemic and continue to operate after it. Indeed, this will lead to reduce recovery time and enhance service continuity. A recovery plan is essential to ensure successful healthcare services delivery and management (22).

“The Saudi government, especially Public Health Authority, plans of mitigation of activities were essential to prevent more spread of the disease, reduce the chance of a risk happening, and reduce undesired effects. National administrative and health steps applied before and after COVID-19.” (Expert healthcare professional 3)

“Well, Key performance indicators are now well-known during COVID-19 report hourly cases in MOH dashboard. The phenomenology of spread infection is tracked through specific protocols applied by higher authority.” (Expert healthcare professional 2)

“Only official agencies like MOH and MOI were the main resource to keep the channel between the population through daily accurate conference as early as possible. This approved that information is transferred in more effective and reliable way: it is a psychological impact where rumors are void.” (Expert healthcare professional 5)

The conclusion of the semistructured interviews indicated the effective application of healthcare measures in the pre-pandemic, during a pandemic, and post-pandemic times. Table 4 shows the conclusion of the semistructured interviews among the five expert healthcare professionals in the healthcare system.

**Table 4:** Expert healthcare professionals theme summary of the semistructured interviews of strategic initiatives toward COVID-19 management.

**Domain**	**Subcategory examples**
Health guidelines	Patient safety- access to healthcare
Simulations adaptation	Virtual clinics- technology- education-application
Continuity of services	Response and mitigation- reporting protocols- effective communications

**Figure 1:**
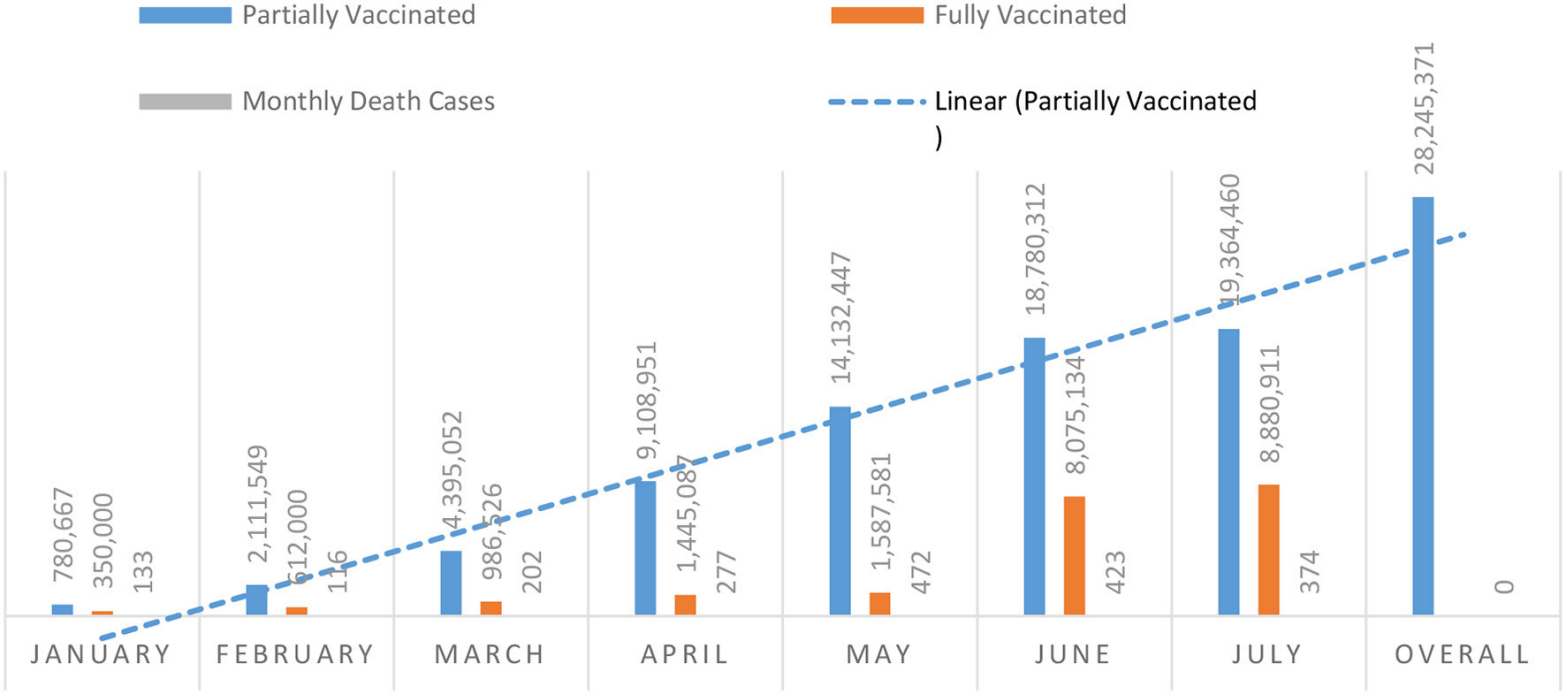
Partially vaccinated and fully vaccinated COVID-19 individuals since 2021 in KSA.

The relationship between different facilities at different levels was not well-organized, and there was a lack of clear channels of communication or policies for sending patients from one specialized hospital to a primary healthcare or a public hospital (23). Conversely, the privatization of healthcare services was frozen to confront the crisis. The number of individuals who were fully or partially vaccinated up till July 2021 is stated in Figure 1.

However, compared to other countries, fully vaccinated individuals in KSA reached almost 25% of the population by the second half of 2021. COVID-19 control measures were developed by the higher authority in KSA including MOH and MOI to primarily alleviate pain from the population regardless of ethnicity, gender, and nationality. The subgoals of this plan were to control and prevent COVID-19 from getting more complicated and out of control.

## Discussion

This research explored key different materials collected from various official resources to trace the macro and micro measurements applied by public organizations in KSA during COVID-19 management. In addition, this research study invited expert healthcare professionals who could contribute to illustrating the risk-reduction on the micro levels. Basically, a policy triangular framework is rooted in a transitional period of both politics and economics. Overall, elements like actors, content, context, and process were represented in this study by healthcare entities, crisis management of COVID-19, and the public healthcare performance, respectively (12). Indeed, this framework has influenced the healthcare policy to outperform and innovate more than what the healthcare system used to Gilson and Raphaely (24). Moreover, KSA was among the early countries to mitigate the impact of COVID-19 even before the first case was reported. This was achieved by the implementation of early precautionary measures learned from past outbreaks, such as, the MERS and the SARS which are both coronaviruses.

In Saudi Arabia, public policy strategy toward the management of COVID-19 has approved its effectiveness to prevent the spread of such a disease within its scope through diverse sectors (4). On a micro level, for example, two major organizations were responsible directly for the clinical and administrative roles within this unprecedented pandemic, confronting a paradox in interrelated authorities (15, 25). The mission of each entity was to seek overall high performance and authority. This activity is relatively higher than the normal procedure to confront sudden national hazards. Also, previous studies from other countries reported security enforcement rather than clinical involvement. For example, healthcare system effectiveness and independence were the most prominent characteristics associated with the Saudi healthcare system, and this is a major requirement according to many international standards including the Institute of Medicine recommendations (3, 26).

There are some possible explanations for the control of only two entities in this crisis. First, the Saudi healthcare system is well-established politically where irrelevant entities cannot penetrate the rules and regulations easily (27). Perhaps most of the democratic states faced reluctant measure enforcements to confront such a crisis (28). Second, the organizational culture is based on the public socialization process where Saudi people acquire their beliefs, values, and attitudes of the culture in which they find themselves independently. Assumingly, such behaviors have been adopted through experience within specific social and cultural nature (3). Moreover, transparency of applying extensive measures increased confidence of the people in healthcare leadership as there was a linear indication in the world report (29).

The decrease in the number of death cases with a decrease in overall cases might be associated with the recent policy implemented by the government. Medical guidelines definitely strengthen hospital capabilities to tackle concurrent and future health challenges. Indeed, effective disease surveillance and response are key performance indicators to maintain a strong healthcare system, especially through leadership and a political system (19). Although KSA has imposed lockdown and other strict measures, simulation (telecommunication/telehealth) was used widely to deliver effective education, wide information, and predict the progress of the outbreak and its possible end (21). In addition, the pandemic generated challenging opportunities for creating a new value: continuity of services. Healthcare service continuity has preserved the value of health although risks were surrounded, by applying public-private partnerships and financial stimulation (30). The conclusion indicated that the government, especially the public health authority, is still committed to providing updated regulations, technical support, and sustainable services for the population through multisectoral collaboration and coordination mechanisms among various entities (20).

While avoiding potential financial consequences, needs of patients were the center of the Saudi healthcare system. As indicated earlier, the medical services are provided free of charge and all individuals (citizens, non-citizens, and illegal residents) were exempted from any financial requirements related to COVID-19 (8). Although illegal residents represent a small portion of the country, yet they add an extra burden on the whole healthcare system. Consequently, this was a stimulus to avoid infections and increase early detection of COVID-19 cases. Thus, the inability to withstand the effects of a hostile environment was eliminated, and people were confident that they will be equally treated, and they would be covered from potential risks and would be able to access healthcare services without spreading the disease among others (31). In simple, risks posed to patient safety were mitigated using patient-specific risk management strategies such as free-public health services, non-financial liabilities, and meeting the expectations and needs of patients.

Limitations of this study include the short period of conducting the cross-sectional study. In addition, there was a probability of bias toward having only one non-Saudi expert healthcare professional. In the absence of an agreed-on definition of what successful crisis management is, no clear assessment application was traced to determine the success of COVID-19 crisis management globally. One common successful method in the healthcare system is to reduce mortality and morbidity rates. However, the current research was designed to reduce ambiguity and to tailor the uniqueness of crisis management to be a good practice.

## Conclusions

In this study, we applied mixed methods to gain insights into the methods applied by the Saudi healthcare agencies adopted to minimize risks and ensure continuity of healthcare services within the Saudi healthcare system.

## Data Availability Statement

The raw data supporting the conclusions of this article will be made available by the authors, without undue reservation.

## Ethics Statement

The studies involving human participants were reviewed and approved by Health Administration. The patients/participants provided their written informed consent to participate in this study.

## Author Contributions

All the authors substantially contributed to the conception, drafting, and approving this manuscript.

## Conflict of Interest

The authors declare that the research was conducted in the absence of any commercial or financial relationships that could be construed as a potential conflict of interest.

## Publisher's Note

All claims expressed in this article are solely those of the authors and do not necessarily represent those of their affiliated organizations, or those of the publisher, the editors and the reviewers. Any product that may be evaluated in this article, or claim that may be made by its manufacturer, is not guaranteed or endorsed by the publisher.
